# Evaluating the Effectiveness of Risk Categories for Prioritising Patients for Best Possible Medication History Completion at a Quaternary Hospital

**DOI:** 10.1177/00185787251365525

**Published:** 2025-08-28

**Authors:** Marissa Ryan, Jane Dunsdon, Karl Winckel, Kate Ziser, Linh Phi, Manae Tominaga, Elizabeth Currey, Nazanin Falconer, Centaine L. Snoswell

**Affiliations:** 1 Princess Alexandra Hospital, Brisbane, QLD, Australia; 2 The University of Queensland, Brisbane, Australia; 3 Queensland University of Technology, Brisbane, Australia

**Keywords:** pharmacist prioritisation, risk criteria, best possible medication history, pharmacist clinical review, high risk patients

## Abstract

**Background:**

The Pharmacist Workload Prioritisation Work Instruction (PWPWI) was developed to optimise clinical pharmacy services, such as best possible medication history (BPMH) completion. Inpatients are assigned a risk category and associated BPMH completion timeframe.

**Aim:**

To determine the proportion of inpatients who met criteria for urgent, high, moderate, and low risk, and the proportion in each category who were reviewed within 24 hours of admission, to inform PWPWI updates.

**Method:**

Clinical, pathologic, medication data, and whether or not the BPMH was completed within 24-hours, was retrospectively collected for inpatients from a single institution and the PWPWI was used to assign risk category.

**Results:**

Data was collected for 280 patients. Prioritisation risk categories were assigned as 3% urgent and requiring immediate review, 61% high risk requiring review within 24-hours, 2% moderate risk requiring review within 48-hours, and 34% low risk. Overall, BPMHs were completed within 24-hours for 54% patients; 50% of the urgent risk individuals, 57% of the high risk, 100% of the moderate risk, and 46% of the low risk.

**Conclusion:**

This study found that nearly two-thirds of patients were urgent or high risk, affecting the completion timeframes. The study’s findings, including four key recommendations, will update the PWPWI. Regular evaluations of such tools are suggested to adapt to changes in clinical care and local context. Following the update, the pharmacy department will receive training to optimise BPMH prioritisation.

## Introduction

Inpatient medication harm results in significant morbidity, mortality, and health care costs. It is estimated to cost AUD 1.4 billion, and USD 30 billion annually, and contributes to a longer length of hospital stay and a poor patient experience.^1,2^ Hospital pharmacist activities, encompassing timely best possible medication history (BPMH), medication reconciliation and review,^1^ reduce medication harm, the cost of care and improve the patient experience.^2^ The ageing population and an increase in medicines for many conditions has led to greater hospital throughput and increasingly complex medication regimens.^3^ Effective prioritisation of patients for BPMH, medication reconciliation and review is essential to ensure optimal use of available pharmacist resources as it allows pharmacists to focus on the highest risk individuals first.^4,5^ Clinical pharmacists provide a variety of important services to inpatients including medication supply, patient education and review of medication regimens for new and existing patients. Patient prioritisation is particularly important in settings with limited resources and high patient volumes, where efficient use of time and expertise can significantly impact patient outcomes.

Existing research has highlighted the potential benefits of patient prioritisation tools in hospital pharmacy services.^4^ Studies have shown that use of prioritisation tools can assist pharmacists in effective identification of high risk patients preventing potential medication errors and medication harm.^6,7^ These tools incorporate a range of criteria such as medication regimens, patient comorbidities, and the potential for drug interactions.^6,7^

With the rapid implementation of digital hospitals, studies have demonstrated that electronic prioritisation tools can enhance workflow efficiency by providing real-time data, which helps in making informed decisions quickly.^6^ However, many existing tools lack external validation (eg, in different hospitals and clinical settings), and have variable performance metrics (eg, specificity and sensitivity).^6,8^ Additionally, for successful implementation of a tool, local clinician buy-in is important^9^ as different pharmacists prioritise patients within their context in different ways.

Criteria to clinically prioritise patients are informed by a variety of sources, including published evidence on the main risk factors for patient harm.^5,7^ However, these published criteria require local consensus, validation and to be tailored to the local context. The Pharmacist Workload Prioritisation Work Instruction (PWPWI) at this quaternary level hospital was developed to incorporate local consensus and context into a tool that would improve patient safety and optimisation of services.

The aim of this study was to determine the proportion of inpatients meeting criteria for the four risk categories of the PWPWI at the study hospital. A secondary aim was to determine whether pharmacists were completing the BPMH in the expected timeframes for the four risk categories, to inform updates to the PWPWI. With increasing acuity of patients coupled with a higher turnover of inpatients, it was important to audit the tool with potential to update the PWPWI. This research will assist with confirming the risk categories and inform the development of a digital version of the PWPWI. Further, Clinical Pharmacy Practice Standards were recently updated in Australia in 2024, which further supported the need to review the PWPWI.^10^

## Method

This study used a retrospective random sample of inpatients at the study hospital to investigate the pragmatic usefulness of the PWPWI. Usefulness was gauged by examining the proportion of inpatients in each category and whether or not they were seen by a pharmacist in the specified timeframe. Ethics approval was received locally.

### Local Tool Development

The prioritisation tool (Table 1) was developed locally at the study hospital and has been in use for 6 years. It was developed by drawing from national and international literature,^5,7^ professional guidelines,^10^ and local prescribing guidelines.^11^ Consensus was reached by gathering senior pharmacists regarded as subject matter experts in pharmacy clinical who reviewed the current and ideal prioritisation processes at the study institution. This happened across a series of meetings to discuss and rank risk factors for assigning risk categories for patient prioritisation for BPMH completion. An initial list of risk factors was compiled from those informed by published evidence, including a national pharmacist survey on the key risk factors for medication harm for patient prioritisation which outlined patient factors (eg, age, length of stay)^5^ and clinical factors (eg, use of high risk medicines, laboratory test results)^5^ and a tool for identifying inpatients at risk of medication-related problems.^7^ The list was refined iteratively until four risk categories emerged, and for each of these risk categories a timeframe during which it was deemed appropriate to complete a BPMH was stipulated (Table 1). The final tool was called The Pharmacist Workload Prioritisation Work Instruction (PWPWI), and it was reviewed by the Pharmacy Department’s Clinical Senate group and operationalised in 2019.^12^ Risk categories and their associated expected BPMH completion timeframes were:

- *Urgent risk,* review immediately.
- *High risk,* complete within 24 hours.
- *Moderate risk,* complete within 48 hours.
- *Low risk,* review twice weekly.

**Table 1.** Risk Categories for Prioritising Best Possible Medication Histories for Inpatients.^12^

Urgent risk = Complete immediately^a^	
International normalised ratio (INR) >5	Activated partial thromboplastin time (APTT) >200 s
Digoxin level >2 ng/mL	Phenytoin level >20 mg/L
Newly initiated or newly admitted clozapine patients (in last 2 d)	Intensive Care Unit (ICU) patients stepping down to other wards (not previously reviewed by ICU pharmacist)
Aminoglycosides intravenous if: – Duration >48 h OR estimated glomerular filtration rate (eGFR) <40 mL/min	Patients with potassium level >6.0 mmol/L or <2.5 mmol/L
High risk = Complete within 24 h	
Medications (either pre-admission OR post-admission: • Any Insulin except monotherapy with insulin glargine • Fentanyl patch or regular oxycodone >40 mg daily OR hydromorphone >10 mg daily • When required, intramuscular antipsychotics being given • Cancer chemotherapy • Intravenous unfractionated heparin or direct oral anticoagulants (full dose) • Enoxaparin >40 mg daily • Antimicrobials where infectious diseases team approval is required (for antimicrobials that are either high cost or have a high potential for resistance) • Parkinson’s disease medications	All patients no pharmacist reviewed for >5 d Patient in whom medication misadventure/adverse drug reaction is a known or suspected cause of admission Patients prescribed methadone/buprenorphine as opioid replacement Patients with a nasogastric/percutaneous endoscopic gastromy (PEG) tube Presentation(s): • Acute Kidney Injury • Sepsis • Patients listed on the emergency board for surgery within 72 h, to ensure appropriate management of medicines prior to surgery • Patients that have had complications during their admission (in last 2 d): ○ Acute behavioural disturbance management (ABDM) powerplan prescribed ○ Renal replacement therapy during admission ○ Fall
Abnormal pathology (in last 2 d) • Estimated glomerular filtration rate (eGFR) <30 mL/min or 30% drop • Potassium 2.5-3.0 or >5.5-6.0 mmol/L • Sodium <125 mmol/L • Platelet drop >50% or platelets <50 × 10^9^/L • White cell count (WCC) <2 × 10^9^/L • Neutrophils <1 × 10^9^/L • Haemoglobin <80 g/L or 25% drop • Activated partial thromboplastin time (APTT) 150-200 s • Blood glucose level <4 mmol/L or >20 mmol/L International normalised ratio (INR) >4.0	
Moderate risk = Complete within 48 h	
Patients aged >85 y of age with multiple comorbidities	Patients on intravenous or oral antibiotics (except where in high risk category above)
Any patients who are outliers on the ward who would otherwise be green (moderate to low priority)	Patients with the following (in last 2 d) • Potassium 3.1-3.5 mmol/L or 5.0-5.5 mmol/L • Magnesium <0.5 mmol/L • Phosphate <0.5 mmol/L • Blood glucose level 15-20 mmol/L
Low risk = Review ONE to TWO times a week	
All remaining patients	

a Once urgent issues are resolved re-assess risk and review as per adjusted priority.

### Patient Selection

Patients included in the study were adult inpatients who were admitted overnight for a minimum of one night before being discharged from the study hospital wards on a Tuesday or Saturday in July, and a Wednesday or Sunday in September 2024. A weekday and weekend discharge day were selected in two different seasons to avoid bias from seasonal factors, admission date, or time of day. Patients admitted to the sleep lab were excluded as they do not routinely receive pharmacist review. No other eligibility or exclusion criteria were applied to patients.

### Data Collection and Analysis

The clinical, pathologic and medication information was manually collected via retrospective chart review during October 1st to November 30th 2024, from the integrated electronic medical record (ieMR) and stored and analysed using a password protected Microsoft Excel file. The current prioritisation tool used at the study hospital was then used to identify which risk category the patient met. Two of the high risk criteria, suspected medication misadventure and suspected adverse drug reaction were not included as part of criteria considered, as information pertaining to these is commonly recorded in the Pharmacy Care Organiser (PCO), a section in the ieMR used for handover notes and other temporary information or the medical admission note. Due to the retrospective nature of the this study, PCO data was not available for capture and medical admission notes were not reviewed due to resource limitations for data collection.

Patient eligibility and the expected associated BPMH completion timeframe within each risk category were recorded. The completion time was defined as the time from admission to completion of a BPMH, which is locally documented by the completion of a Pharmacist Admission Note (PAN). In this study, the definition of BPMH was inclusive of medicines reconciliation.

## Results

Data was collected from every third patient from each weekday and weekend ward list until the target study patient number of 280 inpatients in total was reached. Of the 280 patients, 78% were admitted on weekdays, 46% were medical admissions and 54% were surgical patients. Most of the patients were aged either 41 to 64 years (36%) or 65 to 84 years (37%), with the mean age being 59. There were 194 patients who had risk factors, half (50%) had 1 to 3 risk factors, while lower proportions had 4 to 6 (15%) and very few had 7 to 8 risk factors (1%). The remaining 96 patients (34%) did not have any risk factors, and were therefore categorised as low risk (Table 2).

**Table 2.** Patient Demographic Information Sample Reviewed and Risk Factor and Risk Category Information (n = 280).

Item	Categories (risk level)	Number of patients (%)
Admission day	Weekdays	219 (78)
	Weekend	61 (22)
Gender	Female	110 (39)
	Male	170 (61)
Age (years)	0-18	7 (3)
	19-40	47 (17)
	41-64	102 (36)
	65-84	104 (37)
	>85+	20 (7)
Ward type	Medical	129 (46)
	Surgical	151 (54)
Number of risk factors identified	0	96 (34)
	1-3	138 (50)
	4-6	43 (15)
	7-8	3 (1)
Risk category assigned	Urgent risk	8 (3)
	High risk	171 (61)
	Moderate risk	5 (2)
	Low risk	96 (34)
BPMH completed within 24 h	Urgent risk	4 (50)
	High risk	98 (57)
	Moderate risk	5 (100)
	Low risk	44 (46)
	Total cohort	151 (54)
Frequency of patient flagged with high or urgent risk medications (risk category indicated in parenthesis)	Antimicrobials (high)	73
	Direct-acting oral anticoagulants (DOACs) (high)	33
	Insulin (high)	19
	Intravenous unfractionated heparin (high)	16
	Systemic anticancer therapy (high)	15
	Regular oxycodone >40 mg daily (high)	5
	Parkinsons disease medication (high)	4
	Regular hydromorphone >10 mg daily (high)	3
	Fentanyl patch (high)	2
	Aminoglycosides IV >48 h (urgent)	2
	Aminoglycosides and eGFR <40 mL/min (urgent)	1
	Enoxaparin >40 mg daily (high)	1
	Opioid treatment program (high), acute behavioural disturbance management (ABDM) powerplan initiated (high), clozapine (urgent), when required intramuscular antipsychotics (high)	0
Frequency of patients flagged with the pathology results (risk category indicated in parenthesis)	eGFR <30 mL/min (high)	23
	Potassium >6 mmol/L or <2.5 mmol/L (urgent)	5
	Potassium 2.5-3.0 or >5.5-6.0 mmol/L (high)	9
	Potassium 3.1-3.5 mmol/L or 5-5.5 mmol/L (moderate)	28
	Haemoglobin <80 g/L (high)	7
	Platelet <50 × 10^9^/L (high)	6
	Blood glucose level <4 mmol/L or >20 mmol/L (high)	6
	Blood glucose level 15-20 mmol/L (moderate)	6
	Magnesium <0.5 mmol/L (moderate)	2
	Phosphate <0.5 mmol/L (moderate)	2
	White cell count (WCC) <2 × 10^9^/L (high)	2
	Neutrophils <1 × 10^9^/L (high)	2
	Activated partial thromboplastin time (APTT) >150 (high)	2
	Sodium <125 mmol/L (high)	1
	International normalised ratio (INR) (high)	1
	Digoxin >2 mg/mL (urgent), phenytoin >20 mg/mL (urgent)	0
Frequency of patients flagged with case mix factors (risk category indicated in parenthesis)	Ward outlier (moderate)	27
	Fall (high)	24
	Sepsis (high)	22
	Electronic surgery (e-surge) board listed patients (high)	20
	Acute kidney injury (high)	14
	Renal replacement therapy during admission (high)	13
	Nasogastric tube (for feeding) (high)	12
	>85 y with multiple co-morbidities (high)	11
	PEG tube (high)	4

Regarding the distribution of patients across the risk categories, 3% were urgent risk requiring immediate pharmacist review, 61% were high risk, 2% moderate risk, and 34% low risk.

When the reasons for being prioritised as high risk were examined, four main domains emerged; high risk medications prescribed, high risk pathology results, case-mix factors, and BPMH not completed within 7 days. The most common high risk medications within the overall cohort were antimicrobial agents that required Infectious Diseases approval (n = 73), direct-acting oral anticoagulants (DOACs) (n = 33), and insulin (n = 19). While the most common pathology results that were identified included eGFR <30 mL/min (n = 23), and potassium levels out of range (n = 5 urgent, n = 9 high, n = 18 moderate). Of note, in the group studied, there were no cases of patients who were prescribed when required intramuscular antipsychotics, opioid treatment program clients, clozapine, or powerplans (a type of digital protocol) for acute behavioural disturbance management were observed. There were also patients who were categorised as high risk due to case-mix factors such as having a history of falls (n = 24), or documented sepsis in their initial 24-hours of admission (n = 22).

To explore the types of risk factors and their impact on overall risk rating for prioritisation purposes, a sub-set of the individuals with a single risk factor who were not low risk was examined (n = 41, 43% of single risk patients). They were designated a prioritisation risk level of urgent, high, or moderate based on their single risk factor (Table 3). The most common risk reason was a prescribed antimicrobial that required special approval from the infectious diseases medical team (n = 8, 20%), prescribed DOACs (n = 6, 15%), and patients who had nasogastric (NG) tube (n = 5, 12%).

**Table 3.** Details of Patients with One Risk Factor (n = 41).

Risk factor (risk category indicated in parenthesis)	Number of patients (%)
Antimicrobials (high)	8 (20)
Direct-acting oral anticoagulants (DOACs) (high)	6 (15)
Nasogastric tube (for feeding) (high)	5 (12)
Oxycodone >40 mg daily (high)	4 (10)
Fall (high)	4 (10)
Ward outlier (moderate)	4 (10)
Potassium >6 mmol/L or <2.5 mmol/L (urgent)	2 (5)
Potassium <2.5 to 3.0 mmol/L or >5.5 to 6.0 mmol/L (high)	1 (2)
Intravenous unfractionated heparin (high)	3 (7)
Parkinsons disease medication (high)	1 (2)
Acute kidney injury (high)	1 (2)
Electronic surgery (e-surge) board listed patient (high)	1 (2)
>85 y with multiple co-morbidities (high)	1 (2)

Finally, BPMHs for the 280 patients were surveyed to determine the number completed within 24 hours. Additionally, the proportion of BPMHs completed for moderate risk patients within 48 hours and low risk patients within 72 hours, was investigated. BPMHs were complete for 50% of urgent patients (n = 4 of 8), 57% of high risk patients (n = 98 of 171), 100% of the 5 moderate risk patients, and 46% of low risk patients (n = 44 of 96). For moderate risk patients, 100% (n = 5) were completed in 48 hours, and for low risk patients, only an additional 4% (n = 4 of 96) of BPMHs were completed between the 24 to 72 hours timeframe, giving a total of 50% (n = 48 of 96) completed within 72 hours.

## Discussion

This study identified that 61% of patients at our hospital were categorised as ‘high risk’ and 3% as ‘urgent’ for immediate review and intervention based on locally developed patient prioritisation criteria. The top three risk criteria contributing to a high-risk categorisation were the use of high-risk medications, pathology results, and case-mix factors.

The high percentage of high risk patients may reflect the quaternary nature of the hospital, with increasing patient acuity (eg, patients on anti-cancer therapies or those who have undergone solid organ transplants) and an aging population with multiple comorbidities. Frequent infections requiring antimicrobials and chronic conditions necessitating DOACs also contributed to the high-risk categorization. The lower-than-expected prevalence of urgent and moderate-risk patients suggests that the tool could be refined to better distribute patients across risk categories. Given that patients had an average of two or more high-risk criteria, and 16% had four or more, future iterations of the tool should focus on a weighted scoring system combining multiple risk factors, as described by Falconer et al.

### Comparison with Existing Prioritisation Tools

The literature describes two approaches for identifying and prioritising patients for hospital pharmacist review: consensus-based criteria or tools, and prediction models. Consensus-based tools, like our criteria, often include polypharmacy, high risk medications (eg, insulin, anticoagulants, opioids, antipsychotics), and laboratory markers (eg, deteriorating renal function, and out of range electrolyte levels). These criteria were also included in our checklist and commonly contributed to patient risk. However, there is limited evidence of their ability to mitigate medication harm and further validation studies are needed. A recent systematic review suggests that they are useful for identifying errors and optimisation of clinical workflows for pharmacists.^13^

Prediction models have been developed and validated for inpatient prioritization and discharge services,^14^ incorporating similar clinical criteria with variable predictive ability and accuracy. Further work is needed to test the clinical and workflow impact of these models in practice. Both methods require adaptation to local health service needs and iterative optimisation.

### How Should You Determine an Optimal Risk Threshold?

Previous research suggests different approaches to determining risk thresholds for prioritizing patients for clinical pharmacist services. Retrospective data analysis using historical patient data can determine at what scores key outcomes, such as medication errors or harm, occur. Statistical methods like receiver operating characteristic curve analysis (ROC AUC) are frequently used in predictive modelling tools.^15^

An equally acceptable approach for criteria-based tools is to ensure that the number of patients flagged as high risk aligns with clinical priorities and departmental needs. Previous research highlights that it is reasonable to flag approximately the top 20% to 30% of patients as high-risk.^5,16-18^ Overall, these results demonstrate that the PWPWI tool is sensitive as it identifies patients with risk factors, however its specificity should be studied further due to the high proportion who were designated in the high risk category. Given that 61% of patients currently meet at least one high-risk factor, we reviewed what proportion would meet multiple criteria (eg, three or more) to determine the number of criteria corresponding to patients at risk. A weighted scoring system may also be considered to rank patients from highest to lowest scores in an effort to make the tool more specific in its usefulness for patient prioritisation.

### Medication Reconciliation Within 24 Hours of Patient Admission

Similar proportions of patients categorised as urgent, high, and low risk had BPMH within 24 hours of admission (54% overall across the four risk categories). This highlights the need for education on the use of prioritisation criteria. Research currently underway within Metro South Hospitals suggest that clinicians would prefer a digital real-time application that fits within the ieMR and clinical workflows. Concerningly, patients assessed as ‘urgent’ were not seen in a timely manner. Local and international clinical pharmacy standards expect all these patients to have a BPMH completed within 24 hours. However, some of these patients may have been weekend admissions when pharmacist resources are limited.

### Limitations

The was a small retrospective study of 280 patient admissions. The dataset included patients admitted for planned surgery who were inpatients for a short period, with some lacking pathology results or pharmacist admission notes, categorising them as low risk. Pathology results were often not taken within 24 hours for patients re-admitted within a short period and therefore could not be considered when prioritizing the patient. Patients could fall under urgent risk on day 2 or 3, but this may not have been captured due to the 24-hour cut-off. Additionally, it is unclear if a safety check occurred for urgent risk patients. These limitations provide an accurate picture of the asymmetric information pharmacists use to make prioritisation decisions. Not all criteria will be readily visible or immediately available within patient data, which may have impacted the risk category a patient was placed in. For moderate risk and low risk patients, it would have been useful to record the time from admission to BPMH to inform the achievability of the review timeframe. Additionally, the e-Surgery board for patients is not retrospectively available for review as it is a temporary and changing board, therefore patients meeting this criteria were only identified retrospectively if this was recorded in the notes.

### Recommendations

The authors agreed on four key recommendations from the study results:

(1) Refine the categorisation process to account for risk factors to be considered cumulatively.

*Rationale*: Given that many patients (n = 194) had risk factors, with 50% having 1 to 3 risk factors, 15% having 4 to 6 risk factors, and 1% having 7 to 8 risk factors, it was not surprising that a significant proportion met the criteria for the high risk category. The group proposes moving all high risk criteria to moderate risk criteria. If a patient has 4 or more risk factors in the moderate criteria, they are then classified as high risk. This will ensure a greater proportion of patients in the updated high-risk category have a greater chance of being reviewed within 24 hours.

(2) Completion of BPMH for low-risk patients within 72 hours.

*Rationale*: Low-risk patients previously did not have a timeframe for completion of BPMH. We suggest patients in this category have a BPMH completed within 72 hours.

(3) Inclusion of First Nations People as part of the high risk category.

*Rationale*: As guided by the literature and known risk factors for poor health outcomes, the criteria should include identifying First Nations People as part of the high risk category to align with state-wide plans to improve access to care for First Nations People.

(4) Clarify the expectations around pharmacist interventions for patients identified as urgent.

These patients are routinely prioritised as urgent for one immediately actionable risk factor that once addressed will allow for prioritisation reassessment. Education should be provided to train pharmacists on the safety screening of a patient’s ieMR to recognise and weigh different risk factors when prioritising patients to allow for appropriate action of urgent risk factors and associated documentation, followed by completion of the BPMH in 24 hours.

## Conclusion

This study successfully evaluated the effectiveness of using a risk categorisation tool to prioritise BPMH completion for inpatients at a quaternary hospital. Achievability of BPMH completion timeframes for the corresponding risk categories was impacted due to nearly two-thirds of the patients being urgent or high risk, and a large proportion of low risk patients being completed within 24 hours. The findings from this study including the four key recommendations will be used to update the PWPWI. Such tools should be evaluated every few years to account for changes to clinical care pathways (eg, medications prescribed) and local context which may impact risk factors and therefore risk category. Once the PWPWI is updated, training and education for the pharmacy department will be carried out to ensure optimisation of BPMH prioritisation for inpatients.
